# Contrasting roles of H3K4me3 and H3K9me3 in regulation of apoptosis and gemcitabine resistance in human pancreatic cancer cells

**DOI:** 10.1186/s12885-018-4061-y

**Published:** 2018-02-06

**Authors:** Chunwan Lu, Dafeng Yang, Maria E. Sabbatini, Aaron H. Colby, Mark W. Grinstaff, Nicholas H. Oberlies, Cedric Pearce, Kebin Liu

**Affiliations:** 1 Department of Biochemistry and Molecular Biology, Medical College of Georgia, 1410 Laney Walker Blvd, Augusta, GA 30912 USA; 2 Georgia Cancer Center, Medical College of Georgia, Augusta, GA 30912 USA; 3 Charlie Norwood VA Medical Center, Augusta, GA 30904 USA; 4 Department of Biological Sciences, Augusta University, Augusta, GA 30904 USA; 5 Ionic Pharmaceuticals, Brookline, MA 02445 USA; 6 Department of Biomedical Engineering, Boston University, Boston, MA 02215 USA; 7 Department of Chemistry and Biochemistry, University of North Carolina at Greensboro, Greensboro, NC 27402 USA; 8 Mycosynthetix, Inc, Hillsborough, NC 27278 USA

**Keywords:** H3K9me3, H3K4me3, Gemcitabine, Chemoresistance, Apoptosis

## Abstract

**Background:**

Pancreas ductal adenocarcinoma (PDAC) has the most dismal prognosis among all human cancers since it is highly resistant to chemotherapy, radiotherapy and immunotherapy. The anticipated consequence of all therapies is induction of tumor apoptosis. The highly resistance nature of PDACs to all therapies suggests that the intrinsic tumor cell factors, likely the deregulated apoptosis pathway, are key mechanisms underlying PDAC non-response to these therapies, rather than the therapeutic agents themselves. The aim of this study is to test the hypothesis that epigenetic dysregulation of apoptosis mediators underlies PDAC resistance to gemcitabine, the standard chemotherapy for human PDAC.

**Methods:**

PDAC cells were analyzed for apoptosis sensitivity in the presence of a selective epigenetic inhibitor. The epigenetic regulation of apoptosis regulators was determined by Western Blotting and quantitative PCR. The specific epigenetic modification of apoptosis regulator promoter chromatin was determined by chromatin immunoprecipitation in PDAC cells.

**Results:**

Inhibition of histone methyltransferase (HMTase) by a selective HMTase inhibitor, verticillin A, significantly increased human PDAC cell sensitivity to gemcitabine-induced growth suppression. Verticillin A treatment decreased FLIP, Mcl-1, Bcl-x and increased Bak, Bax and Bim protein level in the tumor cells, resulting in activation of caspases, elevated cytochrome C release and increased apoptosis as determined by upregulated PARP cleavage in tumor cells. Analysis of human PDAC specimens indicated that the expression levels of anti-apoptotic mediators Bcl-x, Mcl-1, and FLIP were significantly higher, whereas the expression levels of pro-apoptotic mediators Bim, Bak and Bax were dramatically lower in human PDAC tissues as compared to normal pancreas. Verticillin A downregulated H3K4me3 levels at the *BCL2L1*, *CFLAR* and *MCL-1* promoter to decrease Bcl-x, FLIP and Mcl-1 expression level, and inhibited H3K9me3 levels at the *BAK1*, *BAX* and *BCL2L11* promoter to upregulate Bak, Bax and Bim expression level.

**Conclusion:**

We determined that PDAC cells use H3K4me3 to activate Bcl-x, FLIP and Mcl-1, and H3K9me3 to silence Bak, Bax and Bim to acquire an apoptosis-resistant phenotype. Therefore, selective inhibition of H3K4me3 and H3K9me3 is potentially an effective approach to overcome PDAC cells resistance to gemcitabine.

## Background

Pancreas ductal adenocarcinoma (PDAC) is the most aggressive malignancy with the most dismal prognosis among all human cancers. The overall 5-year survival rate of PDAC patients is only 6%, and the median survival time for PDAC patients with locally advanced or metastatic disease, which accounts for over 80% of all human PDAC cases, is about 6–10 months [1]. A major cause of this poor prognosis is lack of effective therapies as PDAC is highly resistant to chemotherapy, radiotherapy and immunotherapy [2–4]. Gemcitabine has been, for years, the standard therapy for PDAC patients. However, gemcitabine only increases the survival rate of PDAC patients with advanced disease by a median duration of 5 weeks suggesting that human PDAC cells are either intrinsically resistant or acquire resistance to gemcitabine [1, 5].

The host immune system plays an important role in pancreatic cancer growth control and progression [4, 6–8]. Cancer immunotherapy has made clinically significant advances in the treatment of human cancers in the last few years. In addition to the first FDA-approved immune checkpoint cytotoxic T lymphocyte antigen-4 (CTLA-4) inhibitor, immune checkpoint programmed death-1 (PD-1) and programmed death-1 ligand-1 (PD-L1) blocking antibodies have been also recently approved for treatment of many types of human cancers. Since 2014, anti-PD-1 and anti-PD-L1 antibodies have been shown to induce objective responses in about 20–30% of cancer patients and many of these responses are durable [9, 10]. However, PDAC stands out as one of the few cancers that do not respond to checkpoint immunotherapy [10–15].

The anticipated consequence of all therapies, including chemotherapy, radiotherapy and immunotherapy, is induction of tumor apoptosis. The highly resistance nature of PDACs to various therapies suggests that the tumor cell intrinsic factor, likely the deregulated apoptosis pathway, rather than the therapeutic agents themselves, is one of the major mechanisms for the non-responsiveness of PDAC to these therapies. Therefore, identification of the deregulated apoptosis mediators and development of a targeted therapy that acts through a different mechanism of action than the currently existing therapeutic agents will have the potential to suppress PDAC growth or overcome PDAC resistance to current therapies.

It is well-known that genetic alterations [16–21] in concert with inflammatory factors in the tumor microenvironment [4, 7, 8] drive pancreatic cancer development. Recent studies, however, have shown that epigenetic changes also promote PDAC through aberrant gene expression patterns [6, 22–25]. The methylation of N-terminal lysine residues in histones H3 and H4 plays a fundamental role in the regulation of gene expression through chromatin structure modulation. Histone methyltransferases (HMTase) catalyze the methylation of histones to modify chromatin structure, thereby influencing gene expression patterns during cellular differentiation and embryonic development [26]. Recent studies have shown a critical role of aberrant HMTase expression in human cancers, including PDAC [22–26]. Importantly, unlike genetic defects, which are permanent and non-reversible mutations in the DNA primary sequence of the cancer genome, epigenetic alterations, including histone methylation, is a reversible process that has made HMTases attractive as molecular targets for novel cancer therapies [26–30]. Furthermore, most of current chemotherapeutic agents target the genetic program of cancer cells. Targeting HMTases may be an effective alternative therapeutic approach for PDACs that are refractory to therapies that target the tumor cell genetic program [31–33].

In previous studies, we have developed the novel HMTase inhibitor verticillin A, which selectively inhibits six HMTases and suppresses 5-FU-resistant colon cancer growth [34, 35]. Here, we showed that verticillin A inhibits HMTases to alter H3K9 and H3K4 methylation. Thereby, it regulates the expression of a number of apoptosis regulatory genes, including Mcl-1, FLIP, Bcl-x, Bak, Bax and Bim, effectively sensitizing human PDAC cells to gemcitabine. Our data suggest that targeting HMTase is an effective approach to alter the apoptosis-resistant phenotype of human PDAC and overcome their resistance to chemotherapy.

## Methods

### Human cancer cells

Human pancreatic cell lines MiaPaCa2 (Cat# CRL-1420), PANC1 (Cat# CRL-1469), CFPAC1 (Cat#CRL-1918) and SW1990 (CRL-2172) were obtained from American Type Culture Collection (ATCC, Manassas, VA). ATCC has characterized these cells by morphology, immunology, DNA fingerprint, and cytogenetics. De-identified human normal pancreas and pancreatic carcinoma tissues were obtained from Georgia Cancer Center tumor bank. The treatment history of these patients is not available. Normal pancreatic tissues were collected from the adjacent normal tissues of the tumor tissues. All studies with human specimens were carried out according to protocol (933148–1) approved by Augusta University Institutional Review Board.

### Cell viability assays

Cell viability assays were performed using the MTT cell proliferation assay kit (ATCC, Manassas, VA) according to the manufacturer’s instructions.

### Western blotting analysis

Western blotting analysis was performed as previously described [36]. Cells were collected and lysed in cytosol buffer [10 mM Hepes, pH 7.4, 250 mM Sucrose, 70 mM KCl, 1.5 mM MgCl_2_, 1 mM EDTA, 1 mM EGTA, protease and phosphatase inhibitor cocktails (Calbiochem, Billerica, MA), and 0.01% digitonin] for 10 min. The supernatant was collected as cytosol fraction. The pellet was then resuspended in mitochondria extraction buffer (50 mM Tris-HCl pH 7.5, 100 mM NaCl, 10mMMgCl_2_, 2 mM EGTA, 2 mM EDTA, 1% NP40, and 10% glycerol), incubated on ice for 10 min and centrifuged at 13,000 RPM for 10 min. The supernatant was collected as organelle-enriched mitochondrial fraction. Tumor tissues and normal pancreatic tissues were homogenized in total lysis buffer (20 mM Hepes, pH 7.4, 20 mM NaCl, 1% glycerol, and 1% Triton x-100) with a tissue homogenizer. Cytosolic and mitochondrial fractions and total tissue lysate were resolved in 4–20% SDS polyacrylamide gel and analyzed by Western blotting. Sources of antibodies are: Bax, Mcl-1, FLIP, Bcl-x and cytochrome C: BD Biosciences (San Diego, CA); Bak, Bid, Bim, cleaved Caspase 8, cleaved Caspase 3, cleaved Caspase 9, and cleaved PARP: Cell Signaling Tech (Danvers, MA); H3K9me3 (Abcam, MA); β-actin: Sigma-Aldrich (St Luis, MO). Detailed antibody information is listed in Table 1.

**Table 1:** Antibodies

Antibody	Source	Cat number	Application
H3K9Me3	Abcam	ab8898	WB,ChIP
H3	Cell signaling technology	4499	WB
FLIPL	Cell signaling technology	3210	WB
Mcl-1	Santa Cruz	sc-819	WB
Bcl-2	BD Biosciences	610539	WB
Bcl-x	BD Biosciences	610747	WB
Bax	Abcam	ab32503	WB
Bid	Cell signaling technology	2002	WB
Bak	Upstate	6536	WB
Bim	Cell signaling technology	2933P	WB
CoxIV	Cell signaling technology	4844	WB
Cleaved caspase 8	R&D system	AF705	WB
Cleaved caspase 9	Cell signaling technology	9501S	WB
Cleaved caspase 3	Cell signaling technology	9661S	WB
Cleaved PARP	Cell signaling technology	9541S	WB
Cytochrome C	BD Pharmingen	556433	WB
β-actin	Sigma	A5441	WB
H3K4me3	Cell signaling technology	9751S	ChIP

### Analysis of H3K9 methylation

Verticilin A was isolated, purified and characterized as previously described [37]. Cells were treated with verticillin A for 2 days and incubated in NETN buffer (20 mM Tris-HCl, pH 8, 150 mM NaCl, 5 mM EDTA, 0.5% NP40, 1.5 mM PMSF, 1.7μg/ml aprotinin, 1mN NaV, 0.5 mM NaF) containing protease inhibitor cocktails (Millipore) for 30 min, centrifuged at 13,000 RPM for 5 min, resuspended the detergent-insoluble pellet in 0.1 M HCl, incubated on ice for 30 min and centrifuge at 13,000 RPM for 10 min. Add 1 M Tris pH 9.0 into the supernatant then do Western Blotting analysis. The blot was probed with antibodies specific for H3K9me3 (Abcam) and anti-H3 (Cell Signaling) which was used as the normalization control.

### Chromatin immunoprecipitation (ChIP) assay

ChIP assays were carried out as previously described [6]. Briefly, cells were crosslinked and fixed and the sheared chromatin fragments were immunoprecipitated using anti-H3K9me3 (Abcam) and anti-H3K4me3 antibody (Cell Signaling). The gene-specific promoter DNA was detected by qPCR using promoter DNA-specific primers as listed in Table 2.

**Table 2:** PCR primer sequences

BCL-x-F	GCACAGCAGCAGTTTGGATGC
BCL-x-B	GAGGATGTGGTGGAGCAGAGAAG
MCL-1-F	TCCCTTTTCCTTGGACTGGTATC
MCL1–1-B	GATGACCTTATGGCTCTGAGATGG
FLIP-F	CGAGGCAAGATAAGCAAGGA
FLIP-B	CACATGGAACAATTTCCAAGAA
MIM-F	TCTGAGTGTGACCGAGAAGGTAGAC
BIM-B	CCGATACGCCGCAACTCTTG
BAK-F	TACCGCCATCAGCAGGAACAGGAG
BAK-B	AAGCCCAGAAGAGCCACCACAC
BAX-F	CCCCCGAGAGGTCTTTTTCC
BAX-B	ATCCAGCCCAACAGCCGCTC
BIM-ChIP-F	GAGGAGGGACGGGGTATTTTG
BIM-ChIP-B	TGCTGGGCTCGCAGATAACC
BAX-ChIP-F	CCTGCCCGAAACTTCTAAAAATGG
BAX-ChIP-B	CCAATGAGCATCTCCCGATAAG
BAK1-ChIP-F	CCCCAATGCGACTACAGAACTG
BAK1-ChIP-B	AGGCAGGAGAATCCCTTGAACC
MCL1-ChIP-F	AACTTCCCCGTCCTCTTCCTTC
MCL-ChIP-B	TTCTCGTGGCTACCTCTGTGCTTC
FLIP-ChIP-F	CCGACGAGTCTCAACTAAAAGGG
FLIP-ChIP-B	AAAGAAACCGAAAGCCTGGAAG
BCL-x-ChIP-F	CTCTCCCGACCTGTGATACAAAAG
BCL-x-ChIP-B	CACCTACATTCAAATCCGCCTTAG

### Gene expression analysis

Normal human pancreas tissues and human pancreatic tumor tissues were homogenized using a tissue homogenizer in Trizol (Life Technologies) to isolate total RNA. cDNA was synthesized from total RNA and used for analysis of gene expression using gene-specific primers (Table 2) in the StepOne Plus Real-Time PCR System (Applied Biosystems). β-actin was used as an internal control.

## Results

### Inhibition of HMTase suppresses human PDAC cell growth

**Fig. 1:**
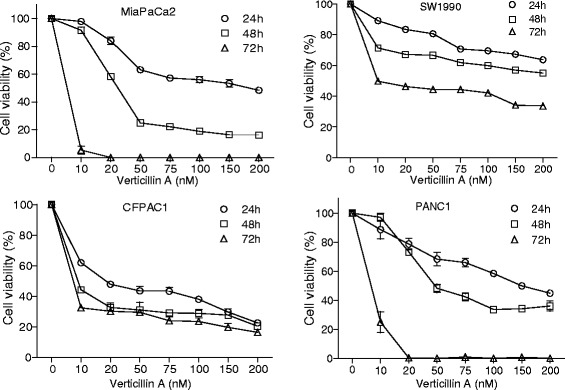
Verticillin A inhibits PDAC cell growth alone. Human PDAC cells were cultured in the presence of various concentrations of verticillin A as indicated. Cell growth was monitored by MTT assay at days 1, 2 and 3. The cell viability of untreated cells were set at 100% and used as reference for the viability of the treated cells

Verticillin A selectively inhibits SUV39H1, SUV39H2, G9a, GLP, NSD2 and MLL1 [35]. These HMTases catalyze the methylation of H3K4 and H3K9 [38–40] and it is known that methylated H3K4 and H3K9 regulate PDAC progression [24, 26]. We therefore reasoned that verticillin A might be effective in suppression of human PDAC cell growth. To test this hypothesis, human PDAC cell lines MiaPaCa2, CFPAC1, SW1990 and PANC-1 were cultured in the presence of various concentrations of verticillin A (0–200 nM) for up to 3 days. Tumor cell viability was measured by MTT assay. Verticillin A exhibited potent inhibitory activity to suppress all four PDAC cell lines in a dose-dependent manner (Fig. 1). Furthermore, the inhibitory level increased with the time of treatment with verticillin A. A three-day treatment completely killed MiaPaCa2 and PANC-1 cells *in vitro* (Fig. 1).

### Inhibition of HMTase overcomes human PDAC resistance to gemcitabine

**Fig. 2:**
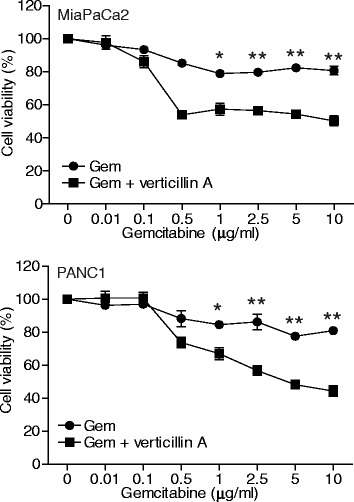
Verticillin A overcomes human pancreatic cell resistance to gemcitabine. MiaPaCa2 and PANC1 cells were cultured in the presence of gemcitabine alone at the indicated concentrations, or in the presence of verticillin A (20 nM) and gemcitabine at the indicated concentrations for 1 day. Cell viability was determined by MTT assay. The cell viability of the control cells (0 μg/ml gembicine) of both the gemcitabine treatment group and the combined gemcitabine and verticillin A (20 nM) were arbitrarily set as 100%. *p < 0.1,**p < 0.01

Most human PDAC cells are intrinsically resistant to chemotherapeutic agents, such as gemcitabine. Indeed, both MiaPaCa2 and PANC-1 cells exhibit minimal sensitivity to gemcitabine at concentrations as high as 10 μg/ml *in vitro* (Fig. 2). Our above observations indicate that verticillin A has potent inhibitory activity against human PDAC cells (Fig. 1). However, verticillin A targets six HMTases and therefore is potentially toxic at high dose in vivo [34]. We therefore sought to test the hypothesis that a sublethal dose of verticillin A is effective in overcoming human PDAC resistance to gemcitabine. We treated human PDAC cells with verticillin A at 20 nM and various concentrations of gemcitabine and analyzed tumor cell viability. Although both MiaPaCa2 and PANC1 cells were highly resistant to gemcitabine, a sublethal concentration of verticillin A significantly increased the sensitivity of both tumor cell lines to gemcitabine (Fig. 2). We therefore conclude that a sublethal dose of verticillin A is effective in overcoming human PDAC cell resistance to gemcitabine.

### Epigenetic regulation of apoptosis regulatory genes

**Fig. 3:**
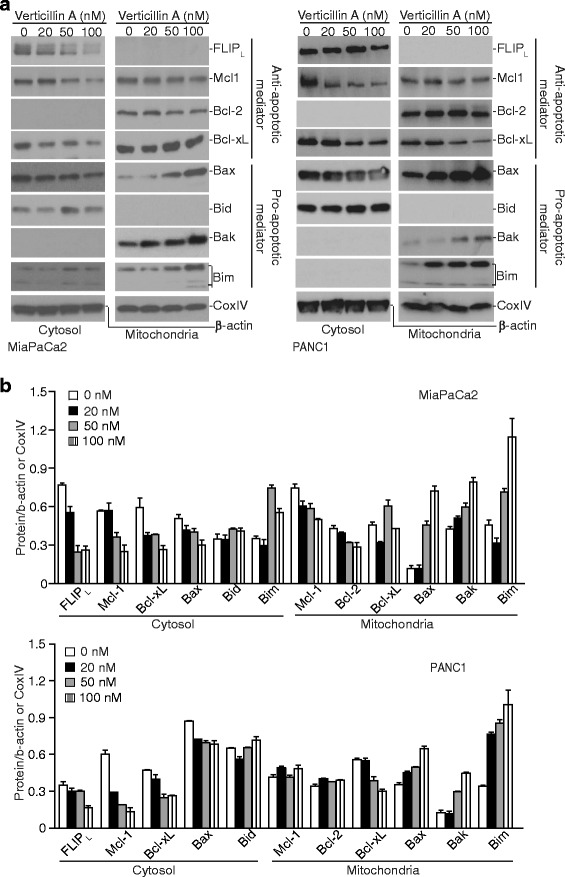
Verticillin A alters the expression levels of a panel of apoptosis regulators. **a** MiaPaCa2 and PANC1 cells were treated with verticillin A at the indicated concentrations for 2 days. Cytosol and mitochondrial fractions were prepared and resolved in 4–20% SDS-polyacrylamide gels, and then analyzed by Western blotting analysis using antibodies for the indicated proteins. β-actin is used as normalization control for the cytosol fractions. CoxIV is used as normalization control for the mitochondrial fractions. Western blots were cropped to improve the conciseness of the results. Gels were run and blotted under the same experimental conditions. Blots from different gels are separated by white space. **b** The protein band intensities were quantified using NIH image J. The cytosol protein levels were normalized as the ratio over the intensity of β-actin. The mitochondria protein levels were normalized as the ratio over the intensity of CoxIV. Column: mean; Bar: SD

HMTase-mediated methylation of H3K4 and H3K9 modulates chromatin structures to play an essential role in gene expression [26]. The above observations that verticillin A effectively suppressed human PDAC cell growth and sensitized the tumor cells to gemcitabine-induced cell growth inhibition suggest that verticillin A may act through regulating the expression of apoptosis regulatory genes. To test this hypothesis, we analyzed the protein levels of the known apoptosis regulatory genes. Western blotting analysis of cytosol and mitochondrial fractions indicated that treatment with verticillin A altered the level of a number of apoptosis regulatory proteins. The pro-apoptotic proteins Bak, Bax and Bim increased, whereas the anti-apoptotic proteins FLIP, Mcl-1 and Bcl-x decreased in the treated cells (Fig. 3a & b). These observations suggested that multiple apoptosis regulatory genes are regulated by HMTase-mediated H3K4 and H3K9 methylation.

### Verticillin A promotes human PDAC cell apoptosis through the intrinsic apoptosis pathway

**Fig. 4:**
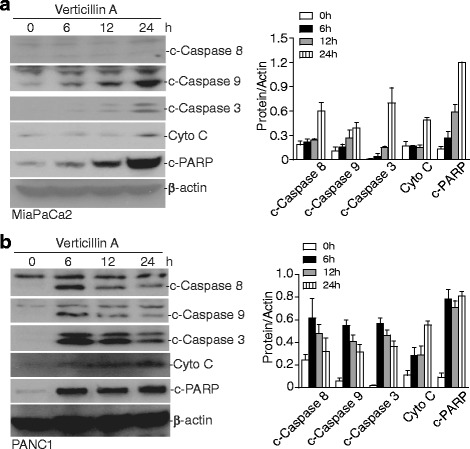
Induction of intrinsic apoptosis. **a** MiaPaCa2 and PANC1 cells were treated with verticillin A (100 nM) for the indicated time. Cytosol fractions were analyzed by Western blotting for the indicated proteins. β-actin was used as normalization control. The protein band intensities were quantified using NIH image J and normalized as the ratio over the intensity of β-actin. Column: mean; Bar: SD. **b** PANC1 cells were treated with verticillin A (100 nM) for the indicated time. Cytosol fractions were analyzed by Western blotting for the indicated proteins. c-Caspase stands for cleaved-Caspase. β-actin was used as normalization control. The protein band intensities were quantified using NIH image J and normalized as the ratio over the intensity of β-actin. Column: mean; Bar: SD

To determine whether the altered apoptosis regulatory protein levels are translated into activation of apoptosis signaling pathway, we then analyzed caspase activation. Western blotting analysis of cytosol fractions indicated that treatment with verticillin A increased the activation of caspases 8, 9 and 3 as indicated by upregulated cleavage of the pro-caspases in both MiaPaCa2 and PANC1 cells. Increased caspase activation was accompanied by elevated cytochrome C release and cleaved PARP (Fig. 4a & b). Therefore, we conclude that inhibition of HMTases alters the expression levels of a set of apoptosis regulatory genes to activate the intrinsic apoptosis pathways.

### Verticillin A inhibits H3K9 trimethylation

**Fig. 5:**
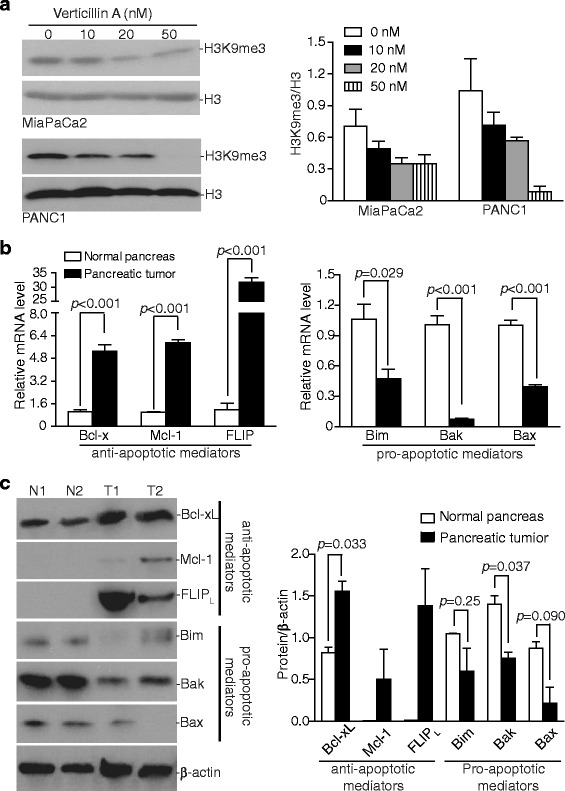
Dysregulation of apoptosis regulatory mediators in human PDAC. **a** MiaPaCa2 and PANC1 cells were treated with verticillin A at the indicated concentrations for 2 days. Acid extraction was prepared from the cells and analyzed by Western blotting with the antibodies specific for the indicated methylated histones. The protein band intensities were quantified using NIH image J and shown at the right panel. The H3K9me3 protein level was normalized as the ratio over the intensity of Η3. **b** RNA was extracted from human pancreatic tumor tissues and adjacent normal pancreas tissues. The mRNA levels of the indicated genes were analyzed by qPCR using gene-specific primer with β-actin as internal control. **c** Total protein was prepared from human pancreatic tumor tissues (patients T1 and T2) and adjacent normal pancreas tissues (N1: adjacent normal tissue from patient T1; N2: adjacent normal tissues from patient T2), and then analyzed by Western blotting analysis using antibodies for the indicated proteins. The protein band intensities were quantified using NIH image J and normalized as the ratio over the intensity of β-actin. Column: mean; Bar: SD

Two of verticillin A targets are SUV39H1 and SUV39H2 [35], which has redundant functions in H3K9 trimethylation [38–40]. We next sought to determine whether verticillin A inhibits H3K9me3 in human PDAC cells. MiaPaCa2 and PANC1 cells were cultured in the presence of verticillin A for 2 days and analyzed for H3K9me3 level. Western blotting analysis indicated that treatment with verticillin A decreased H3K9me3 level in a dose-dependent manner in both tumor cell lines (Fig. 5a). Therefore, verticillin A functions in apoptosis regulation and PDAC cell growth suppression at least in part through inhibition of H3K9me3. Based on our previous observation, verticillin A does not alter the global level of H3K4me3 in MiaPaCa2 cells. However, it significantly reduces the H3K4me3 level on *cd274* promotor region in mouse pancreatic tumor cells [6].

### Apoptosis regulatory mediator profiles in human PDAC

The above data demonstrate that human PDACs are highly resistant to gemcitabine and that the HMTase inhibitor verticillin A modulates the expression of a panel of apoptosis regulatory mediators to promote PDAC apoptosis to suppress tumor cell growth. These observations suggest that dysregulation of apoptosis regulatory mediators might be a major mechanism of PDAC chemoresistance. We next analyzed the mRNA level of the six apoptosis mediators whose expression is regulated by an epigenetic mechanism. RNAs were extracted from normal human pancreas tissues and PDAC specimens and used for analysis of gene expression. qPCR analysis indicated that the expression levels of anti-apoptotic proteins Bcl-x, Mcl-1 and FLIP were significantly higher in PDAC tumor tissues than in the normal pancreas tissues. In contrast, the expression levels of pro-apoptotic proteins Bak, Bim and Bax were significantly lower in the PDAC tumor tissues than in the normal pancreas tissues (Fig. 5b). The Bcl-x, Mcl-1, FLIP, Bim, Bak and Bax protein levels were then analyzed by Western blotting (Fig. 5c). Bcl-xL level is significantly higher in the tumor tissues than in normal tissues. Mcl-1 and FLIP proteins were undetectable in normal pancreas tissues but detected in the pancreatic tumor tissues (Fig. 5c). Bax protein level is significantly lower in the pancreatic tumor tissues as compared to normal pancreas tissue (Fig. 5c). These observations suggested that dysregulated expression of a panel of apoptosis regulatory mediators exists in human PDACs, which leads to the block of apoptosis.

### H3K9me3 directly silences Bak, Bax and Bim expression and H3K4me3 directly activates Bclx, Mcl-1 and FLIP expression

**Fig. 6:**
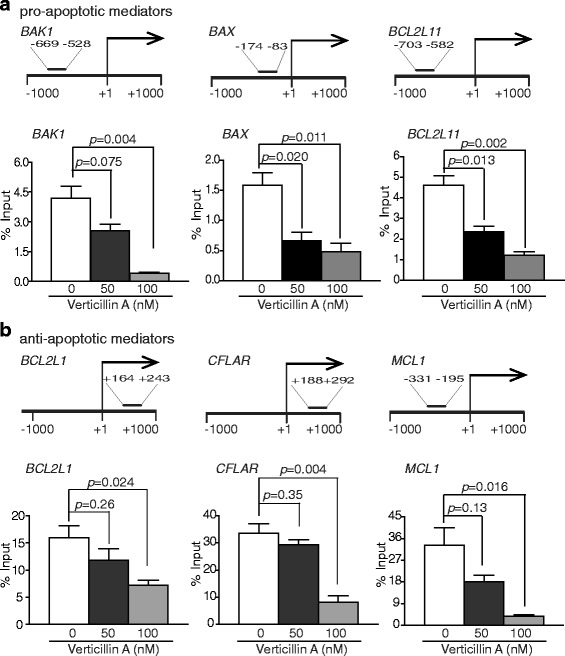
Epigenetic regulation of apoptosis regulatory genes in PDAC cells. **a** MiaPaCa2 cells were crosslinked and subjected to ChIP using H3K9me3-specific antibodies. The levels of H3K9me3-bound genomic DNA were then quantified by qPCR using primers within the − 1000 to + 1000 region relative to the *BAK*, *BAX* and *BCL2L11* (Bim) promotor regions. The ChIP PCR-amplified DNA regions at *BAK*, *BAX* and *BCL2L11* promoter are shown at the top panel. Input genomic DNA was used as internal controls. Column:mean; Bar:SD. **b** Miapaca2 cells were crosslinked and subjected to ChIP using H3K4me3-specific antibodies. The levels of H3K4me3-bound genomic DNA were then quantified by qPCR using primers within the − 1000 to + 1000 region relative to the *BCL2L1* (Bcl-x), *CFLAR* (FLIP) and *MCL1* promotor regions. The ChIP PCR-amplified DNA regions at *BCL2L1*, *CFLAR* and *MCL1* promoter are shown at the top panel. Input genomic DNA was used as internal controls

H3K9me3 is often associated with a transcriptionally repressive chromatin structure to silence gene expression. The observation that Bak, Bax and Bim expression levels are lower in PDAC cells and verticillin A increases Bak, Bax and Bim expression levels suggested that they might be silenced by H3K9me3. To test this hypothesis, we use ChIP assay to analyze the H3K9me3 level at the *BAK1*, *BAX* and *BCL2L11* (gene that encodes for Bim) promoter region. Analysis of immunoprecipitated genomic DNA by H3K9me3-specific antibody indicated that verticillin A indeed decreased H3K9me3 level at *BAK1*, *BAX* and *BCL2L11* promoter region in human PDAC cells (Fig. 6a). In contrast, H3K4me3 is often associated with a transcriptionally active chromatin structure to activate gene expression. The observation that Bcl-x, Mcl-1 and FLIP expression levels are higher in PDAC cells and verticillin A decreases their expression level suggested that they might be activated by H3K4me3. We again used ChIP assay to analyze the H3K4me3 level at the *BCL2L1* (gene that encodes for Bcl-x), *MCL-1* and *CFLAR* (gene that encodes for FLIP) promoter region. Analysis of immunoprecipitated genomic DNA by H3K4me3-specific antibody indicated that verticillin A indeed decreased H3K4me3 level at *BCL2L1*, *MCL-1* and *CFLAR* promoter region in human PDAC cells (Fig. 6b). Taken together, our data determined that dysregulation of a panel of apoptosis regulatory genes by an epigenetic mechanism underlies PDAC chemoresistance and targeting HMTases is an effective means of overcoming human PDAC resistance to gemcitabine.

## Discussion

Gemcitabine is a nucleotide analog that is rapidly metabolized to the active triphosphate form of gemcitabine (2′, 2′-difluoro-2′-deoxycytidine triphosphate, or dFdCTP) once inside the cells. dFdCTP is then incorporated into DNA to stop DNA synthesis by a process known as masked chain termination [41, 42]. Although many cellular responses to dFdCTP incorporation are known, the downstream pathways leading to tumor cell death are largely unknown [43]. However, it has been well-documented that gemcitabine activates caspase-dependent apoptosis signaling pathways in tumor cells [5, 43]. Gemcitabine has been the standard adjuvant therapy for PDAC for the last two decades, but tumor cells are either intrinsically resistant to gemcitabine or develop acquired resistance to gemcitabine within weeks of chemotherapy initiation [1, 5, 21]. To overcome PDAC resistance to gemcitabine, nabpaclitaxel, and the folic acid, fluorouracil, irinotecan, and oxaliplatin (FOLFIRINOX) have been combined with gemcitabine and this combined protocol has shown some improvement in efficacy and survival time compared to gemcitabine alone. Unfortunately, the disease still rapidly advances [1, 5, 25]. Furthermore, although targeted therapies, either alone or in combination with gemcitabine, generate responsiveness in some PDAC patients, the majority of PDAC patients do not respond to targeted therapies or rapidly acquire resistance during therapy [44]. In this study, we observed that the HMTase inhibitor verticillin A dramatically suppresses human PDAC growth. More interestingly, a sublethal dose of verticillin A effectively overcame human PDAC cell resistance to gemcitabine. Verticillin A is a selective HMTase inhibitor that inhibits SUV39H1, SUV39H2, G9a, GLP, NSD2 and MLL1 [35]. These observations suggest that human PDAC cells use deregulation of HMTase to acquire a chemoresistant phenotype.

Verticillin A inhibits PDAC growth at least in part through activating the intrinsic apoptosis pathway as it alters the levels of a panel of apoptosis regulatory genes, mainly the pro-apoptotic Bak, Bax and Bim and the anti-apoptotic Bcl-x, Mcl-1 and FLIP, in human PDAC cells. All these six apoptosis regulatory genes are known to be involved in PDAC growth and progression [45–49]. Analysis of PDAC tissues from human patients revealed that Bak, Bax and Bim are significantly down-regulated, whereas Bcl-x, FLIP and Mcl-1 are notably up-regulated as compared to normal human pancreas tissues. Therefore, it seems that human PDAC cells deregulate multiple apoptosis regulators to confer an apoptosis-resistant phenotype, which may explain the potency of verticillin A in inhibition of PDAC cell growth and in sensitization of tumor cells to gemcitabine-induced growth suppression since it targets all these deregulated genes in the tumor cells. Various inhibitors that selectively target individual apoptosis regulators have been developed and tested in suppression of PDAC growth and progression and show efficacy [45–49]. In light of the observations that multiple apoptosis mediators are deregulated in human PDAC and that the HMTase inhibitor verticillin A simultaneously targets these deregulated apoptosis regulators, it seems that an epigenetic HMTase inhibitors may have an advantage over these selective agents targeting a single apoptosis regulator.

SUV39H1 and SUV39H2 catalyze H3K9me3 [50]. G9a and GLP form a complex to catalyze H3K9Me1 and H3K9Me2 [51]. NSD2 mediates H3K36Me2 [52], and MLL1 catalyzes H3K4me3 [53]. H3K9me3 is often associated with a transcriptionally repressive chromatin and H3K4me3 is often linked to a transcriptionally active chromatin. Consistent with their functions in gene transcription regulation, we observed that verticillin A inhibited H3K9me3 at the *BAK1*, *BAX* and *BCL2L11* promoter chromatin, and H3K4me3 at the *BCL2L1*, *CFLAR* and *MCL-1* promoter chromatin in human PDAC cells. Furthermore, inhibition of H3K9me3 resulted in elevated Bak, Bax and Bim expression level and inhibition of H3K4me3 led to decreased Bcl-x, FLIP and Mcl-1 expression levels in the tumor cells. These observations suggest that PDAC cells use H3K4me3 to increase Bcl-x, FLIP and Mcl-1 expression while using H3K9me3 to silence Bak, Bax and Bim expression, which results in an overall stable apoptosis-resistant phenotype that confers PDAC cell resistance to gemcitabine.

## Conclusions

We determined that a panel of apoptosis regulators was deregulated by epigenetic mechanisms in PDAC cells, suggesting that conventional approach to target individual molecular targets is unlikely to overcome PDAC chemoresistance. Therefore, epigenetic targeting of both H3K4me3 and H3K9me3 is an effective approach to overcome human PDAC resistance to gemcitabine.

## Abbreviations

CTLA4: Cytotoxic T lymphocyte antigen-4
FOLFIRINOX: Folic acid, fluorouracil, irinotecan, and oxaliplatin
HTMase: Histone methyltransferase
PD-1: Programmed death-1
PDAC: Pancreas ductal adenocarcinoma
PD-L1: Programmed death ligand-1
